# Dosimetric impact of orthopedic metal artifact reduction (O‐MAR) on spine SBRT patients

**DOI:** 10.1120/jacmp.v16i5.5356

**Published:** 2015-09-08

**Authors:** Zhilei Liu Shen, Ping Xia, Paul Klahr, Toufik Djemil

**Affiliations:** ^1^ Department of Radiation Oncology Cleveland Clinic Cleveland OH; ^2^ CT Business Unit Philips Healthcare Cleveland OH USA

**Keywords:** metal artifact reduction, CT image reconstruction, dose calculation algorithm, spine SBRT, Monte Carlo

## Abstract

The dosimetric impact of orthopedic metal artifact reduction (O‐MAR) on spine SBRT patients has not been comprehensively studied, particularly with spinal prostheses in high‐dose gradient regions. Using both phantom and patient datasets, we investigated dosimetric effects of O‐MAR in combination of various metal locations and dose calculation algorithms. A physical phantom, with and without a titanium insert, was scanned. A clinical patient plan was applied to the artifact‐free reference, non‐O‐MAR, and O‐MAR phantom images with the titanium located either inside or outside of the tumor. Subsequently, five clinical patient plans were calculated with pencil beam and Monte Carlo (iPlan) on non‐O‐MAR and O‐MAR patient images using an extended CT‐density table. The dose differences for phantom plans and patient plans were analyzed using dose distributions, dose‐volume histograms (DVHs), gamma index, and selected dosimetric endpoints. From both phantom plans and patient plans, O‐MAR did not affect dose distributions and DVHs while minimizing metal artifacts. Among patient plans, we found that, when the same dose calculation method was used, the difference in the dosimetric endpoints between non‐O‐MAR and O‐MAR datasets were small. In conclusion, for spine SBRT patients with spinal prostheses, O‐MAR image reconstruction does not affect dose calculation accuracy while minimizing metal artifacts. Therefore, O‐MAR images can be safely used for clinical spine SBRT treatment planning.

PACS numbers: 87.53.Bn, 87.55.K‐, 87.57.Q‐, 87.57.cp

## I. INTRODUCTION

Metal objects, such as prostheses and dental fillings, can cause artifacts on X‐ray computed tomography (CT) images due to a combination of beam hardening, photon starvation, edge gradient effect, and scatter.^(1)^ These artifacts may obscure visualization of anatomical structures and affect dose calculation accuracy. Various approaches for metal artifact reduction have been developed and can be generally divided into two categories: projection completion and iterative reconstruction. Projection completion methods utilize the filtered back‐projection algorithm and treat the projections passing through the metal as missing projections. The missing projections are then completed by interpolating the measured projections via linear,^(2)^ polynomial,^3^, ^4^, ^5^ or wavelet^6^, ^7^ interpolation techniques. Iterative reconstruction algorithms incorporate various physical models, such as noise^8^, ^9^ and beam hardening,^10^, ^11^, ^12^ into the repetitive image reconstruction process to improve image quality but at the cost of prolonged computational time. Common iterative reconstruction methods include the maximum likelihood expectation maximization algorithm,^13^, ^14^ transmission maximum likelihood algorithm,^(8)^ algebraic reconstruction technique,^(13)^ and alternating minimization algorithm.^(15)^

The first commercial metal artifact reduction algorithm for orthopedic implants (O‐MAR; Philips Healthcare Inc., Cleveland, OH) was released recently.^(16)^ The O‐MAR algorithm has been clinically evaluated for large implants such as hip prostheses, femur rods, and humeral rods.^17^, ^18^, ^19^ Li et al.^(17)^ showed that the O‐MAR algorithm improves CT Hounsfield unit (HU) accuracy, diminishes noise, and reduces metal artifacts (particularly for bilateral hip implants). Their study also demonstrated that the dose distributions calculated on the O‐MAR corrected images were clinically equivalent to those calculated on the uncorrected images with the densities of the artifact regions appropriately overridden.^(17)^ Hilgers et al.^(19)^ also confirmed that the CT number accuracy of the O‐MAR reconstruction was better than that of the non‐O‐MAR reconstruction in a phantom study simulating unilateral and bilateral hip prostheses. Glide‐Hurst et al.^(18)^ investigated the impact of the O‐MAR algorithm on different image bit‐depths (enhanced 16‐bit vs. standard 12‐bit) on patients with hip prostheses or metal rods in extremities. Using Monte Carlo simulation and absolute film dosimetry on phantoms, their study demonstrated that 12‐bit data (both with and without O‐MAR correction) underestimated doses at or near metal implants compared to 16‐bit data.

For patients receiving spine stereotactic body radiation therapy (SBRT), spinal prostheses can be present near the tumors and it is not feasible to completely prevent the radiation beams from passing through the metal implants. In addition, spine SBRT plans often approach the tolerance dose of the spinal cord, so small deviations in dose calculation can impact clinical decision making. Though the O‐MAR algorithm has been shown to reduce metal artifacts and to improve contour delineation, the dosimetric impact of O‐MAR on small implants are not well understood. The purpose of this study was to comprehensively evaluate the dosimetric impact of the O‐MAR algorithm on spine SBRT patients with spinal prostheses.

## II. MATERIALS AND METHODS

### A. Study design

We conducted both phantom and patient studies to investigate the dosimetric effects of O‐MAR in combination of various metal locations and dose calculation algorithms. For the patient study, CT image sets from five patients who received spine SBRT were reconstructed with and without O‐MAR. iPlan (BrainLAB Inc., Westchester, IL) treatment planning systems (TPS) was applied because it was utilized at our institution for treating these spine SBRT patients. Though only pencil beam dose calculation was used clinically, Monte Carlo simulation was included for comparison with pencil beam because Monte Carlo is generally considered as the most accurate dose calculation method. However, iPlan is not capable of overriding density for individual structures, so Pinnacle TPS (Philips Healthcare Inc., Cleveland, OH) was used for the phantom study. We used a CT phantom containing various tissue‐equivalent and titanium inserts with known physical densities. Dose calculation was done with the collapsed cone convolution method from the Pinnacle TPS.

### B. Phantom study

#### B.1 Data acquisition

The phantom study was conducted using a CT electron density phantom (Model RMI 465; Gammex RMI, Middleton, WI). The phantom is made of water‐equivalent materials and contains inserts made of various tissue‐simulating materials and metal. The diameter of each insert is 30 mm, except for titanium (∼13 mm).

With and without a titanium insert, the phantom was scanned twice on a Philips Brilliance Big Bore CT scanner following a clinical spine SBRT protocol at 120 kVp and with 1 mm slice thickness. In the absence of the titanium insert, the CT scan was used as the artifact‐free reference image. On this reference CT image, we purposely overrode the physical density of the solid water insert with that of titanium in the Pinnacle TPS to represent the CT image without artifacts but with a titanium density. With the presence of the titanium insert, the acquired CT images were reconstructed with and without the O‐MAR algorithm.

#### B.2 Analysis of phantom data

**Figure 1:**
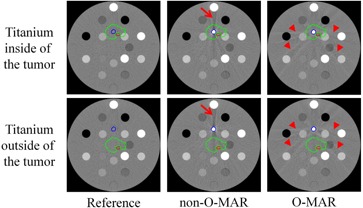
CT images of an electron density phantom with contours mapped from a real patient case. The arrows and arrowheads indicate the metal artifacts. Blue contour=titanium insert,green contour=PTV,red contour=spinal cord.

Three phantom CTs (reference, non‐O‐MAR, and O‐MAR) were exported to the Pinnacle TPS (Version 9.0) in 12‐bit, which is the standard bit‐depth used for most radiotherapy treatment planning. An extended CT density table was generated to include the correct density of titanium ($4.5\;g/{\text{cm}}^{3}$). No manual density override was performed to correct for the metal artifacts in the non‐O‐MAR and O‐MAR phantom CTs. A clinical single‐fraction SBRT plan was then applied to these phantom CTs for dosimetric evaluation. The clinical intensity‐modulated radiation therapy (IMRT) plan was delivered on a Novalis machine (Varian Medical Systems Inc., Palo Alto, CA) using seven 6 MV photon beams at gantry angles of 105°, 130°, 155°, 180°, 215°, 240°, and 265°. The planning target volume (PTV) and organ at risk (OAR) were mapped from the clinical plan to the phantom images with the titanium located either inside or outside of the tumor (Fig. 1). Dose distributions and dose‐volume histograms (DVHs) of the phantom plans were compared. Using OmniPro‐I'm*RT* Software (Version 1.7; IBA Dosimetry America, Bartlett, TN), 2D gamma analysis was performed on phantom plans with and without O‐MAR correction, with criteria of 2% dose difference /2 mm distance to agreement (2%/2 mm) and 1%/1 mm. The low‐dose threshold was set at 10%.

### C. Patient study

#### C.1 Data acquisition

Five patients with spinal prostheses near spinal tumors were retrospectively selected from an institutional review board approved registry. All patients were treated with a single‐fraction SBRT on a Novalis machine using 6 MV photon, for a total dose of 16 Gy (for cervical and thoracic spine) or 15 Gy (for sacrum), following a protocol similar to RTOG 0631.^(20)^ The IMRT plan was designed in the iPlan TPS using seven beams that were approximately evenly distributed from the posterior of the patient. Because the tumors were surrounded by the spinal prostheses, choosing beam angles that avoid the spinal prostheses was not clinically feasible. The PTV (defined as the spinal tumor) and OAR (defined as the true spinal cord) were contoured based on the high resolution T1‐ and STIR‐weighted MRI scans that were fused with the planning CT images. The treatment goal was to deliver the prescription dose to >90% of the PTV. The plan acceptance criteria included that the maximum point dose to the spinal cord was <14 Gy, and <10% of the spinal cord volume received 10 Gy. For the sacrum region, the maximum point dose of the spinal cord was <15 Gy, and <10% of the spinal cord volume received 12 Gy.

#### C.2 Analysis of patient data

Because all initial patient plans were generated with iPlan, the patient plans for this study were evaluated with both the pencil beam and Monte Carlo calculations in the iPlan TPS (Version 4.5). The planning CT of each patient was reconstructed with and without O‐MAR correction. The corrected CT density table including the titanium density of $4.5\;g/{\text{cm}}^{3}$ was used for dose calculation. No manual density override was performed on either the O‐MAR or non‐O‐MAR CTs. The pencil beam algorithm was carried out with a 2 mm dose resolution and with heterogeneity correction. The Monte Carlo calculation was based on the X‐ray Voxel Monte Carlo algorithm.^(21)^ Previous studies demonstrated that this Monte Carlo calculation achieved a good agreement with the measured dose distributions in both homogeneous and heterogeneous media.^22^, ^23^, ^24^, ^25^ The parameters for Monte Carlo dose calculation were set at 2 mm for the spatial resolution, 2% for the mean variance, “dose to medium” for the dose result type, and “accuracy optimized” for the multileaf collimator model.

DVHs and gamma index (2%/2 mm and 1%/1 mm) were generated to compare the dose distributions between the non‐O‐MAR and O‐MAR patient datasets. The differences in selected dosimetric endpoints between the non‐O‐MAR and O‐MAR patient datasets were computed as:

(1) $$difference={\text{Endpoint}}_{O-MAR}-{\text{Endpoint}}_{non-O-MAR}$$

The differences in selected dosimetric endpoints between the pencil beam and Monte Carlo methods were computed as:

(2) $$difference={\text{Endpoint}}_{Monte\;Carlo}-{\text{Endpoint}}_{Pencil\;Beam}$$

The endpoints for PTV were the minimum, maximum, and mean doses (${\text{PTV}}_{-}\min,\;{\text{PTV}}_{-}\max,\;{\text{PTV}}_{-}\text{mean}$), volume receiving the prescribed dose (${\text{PTV}}_{-}V_{100\%}$), and dose that 90% of the target volume received (${\text{PTV}}_{-}D_{90\%}$). The endpoints for the spinal cord were the volume receiving 10 Gy (${\text{CORD}}_{-}V_{10\text{Gy}}$), maximum dose (${\text{CORD}}_{-}\max$), and minimum dose delivered to 0.1 cc (${\text{CORD}}_{-}V_{0.1\text{CC}}$).

## III. RESULTS

### A. Phantom study

#### A.1 Image quality comparison

Figure 1 shows the CT images of the phantom in two scenarios, where the titanium is either inside or outside of the spinal tumor. The reference CT images show no metal artifacts. The non‐O‐MAR images show dark streaks near the titanium insert in the anterior–posterior direction (arrows). The O‐MAR algorithm reduces the original severe artifacts in the anterior–posterior direction, but introduces more dispersed artifacts in other directions (arrowheads).

#### A.2 Dosimetric comparison on phantom data

**Figure 2:**
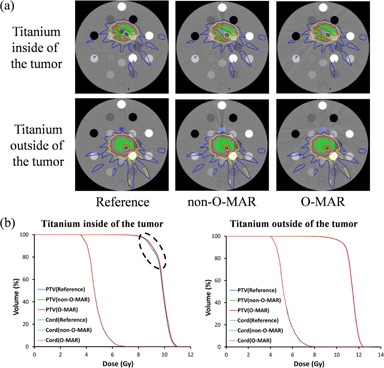
Comparisons of (a) dose distributions and (b) DVHs for a seven‐beam IMRT plan on the phantom data from Pinnacle. Isodose lines: red=10 Gy,yellow=8 Gy,blue=6 Gy. The dashed eclipse indicates the small discrepancy in the DVHs.

Figure 2(a) displays the dose distributions of the seven‐beam IMRT plan on the phantom image sets computed by Pinnacle. Independent of the location of the titanium with respect to the tumor, the dose distributions for the reference, non‐O‐MAR, and O‐MAR image sets were very similar, including the regions with severe metal artifacts. Figure 2(b) shows the corresponding DVHs for the titanium inside and outside of the tumor. Because DVHs did not provide information on the two‐dimensional dose distribution, we also compared the spatial dose distributions using the gamma index of planar dose distributions between non‐O‐MAR and O‐MAR. For the selected plane at the mid‐depth of the inserts, the gamma index was >99.97% (2%/2 mm) or >99.96% (1%/1 mm).

### B. Patient study

#### B.1 Image quality comparison

**Figure 3:**
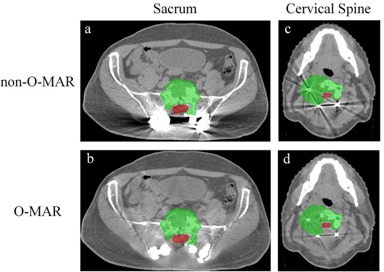
Metal artifact reduction on two representative patient cases from iPlan: (a)‐(b) a case with large metal artifact reduction; (c)‐(d) a case with small metal artifact reduction. Green contour=PTV,red contour=spinal cord.

All five patient cases showed some degree of image quality improvement, ranging from small to large metal artifact reduction. In a sacrum case with large artifact reduction (Fig. 3(a) and (b)), both the streak and darkening artifacts were eliminated after the O‐MAR correction, allowing clear identification of the metal implants and surrounding tissues. In a cervical spine case with small artifact reduction (Fig. 3(c) and (d)), the severity of artifacts was mitigated, but some residual artifacts were still present.

#### B.2 Dosimetric comparison on patient data

**Figure 4:**
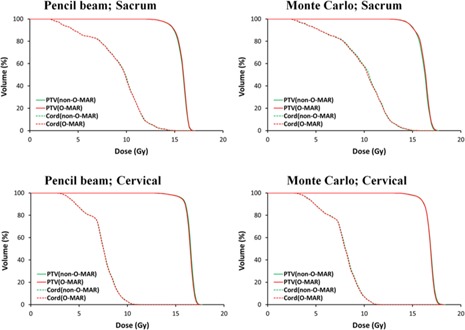
Comparisons of DVHs for the patient data from iPlan.

Figure 4 shows the DVHs of two representative patient cases from iPlan using both the pencil beam and Monte Carlo methods. The DVHs of the PTV and spinal cord were very similar for the non‐O‐MAR and O‐MAR image sets.

**Figure 5:**
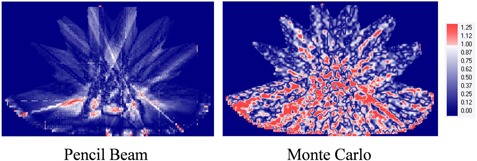
Comparisons of gamma analysis (1%/1 mm) results for the patient data from iPlan. For this patient, the gamma index values were 97.54% for pencil beam and 75.08% for Monte Carlo.

For the five patients, the gamma index (2%/2 mm) of the planar dose distributions at the level of isocenter between non‐O‐MAR and O‐MAR was 99.80%±0.27%(99.33%−100%) for pencil beam and 99.01%±0.47%(98.61%−99.69%) for Monte Carlo. This indicates O‐MAR has minimal effects on dose calculation for these patients with spinal prostheses. When the stricter criteria of 1%/1 mm were used, the gamma index of the planar dose distributions at the level of isocenter between non‐O‐MAR and O‐MAR was 98.14%±1.12%(96.96%−99.93%) for pencil beam and 81.45%±6.18%(75.08%−89.70%) for Monte Carlo. Note the stricter gamma analysis (1%/1 mm) showed greater differences between non‐O‐MAR and O‐MAR, particularly for Monte Carlo method. Figure 5 shows the gamma analysis (1%/1 mm) results of one representative patient case computed by iPlan with both the pencil beam and Monte Carlo methods. Similar to a previous study,^(17)^ there were some dose differences between the non‐O‐MAR and O‐MAR image sets at the beam edges when the pencil beam method was used. The dose differences between the non‐O‐MAR and O‐MAR images sets were randomly distributed when the Monte Carlo method was used, which was likely due to the probability‐based process implemented in Monte Carlo dose calculation.

Table 1 shows the differences in several dosimetric endpoints between the non‐O‐MAR and O‐MAR patient datasets. For the pencil beam method, the differences between non‐O‐MAR and O‐MAR patient datasets were relatively small. For the six endpoints with units of Gy (${\text{PTV}}_{-}\min,{\text{PTV}}_{-}\max,{\text{PTV}}_{-}\text{mean},{\text{PTV}}_{-}D_{90\%},{\text{CORD}}_{-}\max$, and ${\text{CORD}}_{-}D_{0.1\text{CC}}$), the largest difference was observed for ${\text{PTV}}_{-}\max$, where O‐MAR decreased ${\text{PTV}}_{-}\max$ by 0.13 Gy compared to the non‐O‐MAR dataset. For the other two endpoints with units of percentage (%), the largest differences were that O‐MAR decreased ${\text{PTV}}_{-}V_{100\%}$ by 0.9% and increased ${\text{CORD}}_{-}V_{10\text{Gy}}$ by 0.9%, respectively. Nonetheless, the three key clinical dose constraints (${\text{PTV}}_{-}V_{100\%},{\text{CORD}}_{-}V_{10\text{Gy}}$, and ${\text{CORD}}_{-}\max$) were all met when using the O‐MAR dataset, compared to the non‐O‐MAR dataset. The difference between non‐O‐MAR and O‐MAR datasets for the Monte Carlo method were still small, though slightly larger compared to the pencil beam method. The largest differences were that O‐MAR decreased ${\text{PTV}}_{-}\max$ by 0.79 Gy and decreased ${\text{CORD}}_{-}V_{10\text{Gy}}$ by 1.2%. Overall, these results indicate minimal effects from the O‐MAR correction for both the pencil beam and Monte Carlo methods.

Table 2 shows the differences in several dosimetric endpoints between the pencil beam and Monte Carlo methods. For the non‐O‐MAR dataset, the Monte Carlo method significantly increased every endpoint compared to the pencil beam method (p<0.05). The largest differences were that the Monte Carlo method increased ${\text{PTV}}_{-}\max$ by 1.48 Gy and increased ${\text{CORD}}_{-}V_{10\text{Gy}}$ by 14.4%, compared to the pencil beam method. Because the clinical dose constraint for ${\text{CORD}}_{-}V_{10\text{Gy}}$ is <10%, some patient plans would exceed this dose constraint when calculated with the Monte Carlo method, even though they were within the constraint when calculated with the pencil beam. Similar results were observed for the O‐MAR dataset. The largest differences were that the Monte Carlo method increased ${\text{PTV}}_{-}\max$ by 0.81 Gy and increased ${\text{CORD}}_{-}V_{10\text{Gy}}$ by 12.3% compared to the pencil beam method. These results suggest that the pencil beam method underestimates some critical dose constraint (e.g., ${\text{CORD}}_{-}V_{10\text{Gy}}$) for both non‐O‐MAR and O‐MAR datasets, compared to the Monte Carlo method.

**Table 1:** Differences in selected dosimetric endpoints between the non‐O‐MAR and O‐MAR patient datasets (for the pencil beam and Monte Carlo methods, respectively)

*Difference*	*Non‐O‐MAR vs. O‐MAR (Pencil Beam) mean (min, max)*	*Non‐O‐MAR vs. O‐MAR (Monte Carlo) mean (min, max)*
${\text{PTV}}_{-}\min\;(\text{Gy})$	−0.01(−0.08,0.08)	−0.10(−0.60,0.45)
${\text{PTV}}_{-}\max\;(\text{Gy})$	−0.05(−0.13,0.02)	$-0.42{\left(-0.79,-0.03\right)}^{a}$
${\text{PTV}}_{-}\text{mean}\;(\text{Gy})$	−0.02(−0.07,0.04)	0.02 (−0.03,0.06)
${\text{PTV}}_{-}V_{100\%}\;(\%)$	0 (−0.9,0.8)	0.2 (−0.3,0.8)
${\text{PTV}}_{-}D_{90\%}\;(\text{Gy})$	−0.01(−0.03,0)	0.02 (−0.03,0.08)
${\text{CORD}}_{-}V_{10\%\text{Gy}}\;{\left(\%\right)}^{b}$	0.1 (‐0.4, 0.9)	−0.3(−1.2,0.3)
${\text{CORD}}_{-}\max\;(\text{Gy})$	−0.01(−0.04,0.03)	0.02 (−0.20,0.30)
${\text{CORD}}_{-}D_{0.1\text{CC}}\;(\text{Gy})$	0.02 (0, 0.05)	0.04 (−0.03,0.14)

a p−value=0.044.

b ${\text{CORD}}_{-}V_{12\text{Gy}}$ was used for the sacrum case.

**Table 2:** Differences in selected dosimetric endpoints between the pencil beam and Monte Carlo methods (for the non‐O‐MAR and O‐MAR patient datasets, respectively)

*Difference*	*Pencil Beam vs. Monte Carlo (Non‐O‐MAR) mean (min, max)*	*Pencil Beam vs. Monte Carlo (O‐MAR) mean (min, max)*
${\text{PTV}}_{-}\min\;(\text{Gy})$	0.69 (0.28, 1.29)	$0.59{\left(0.14,0.78\right)}^{a}$
${\text{PTV}}_{-}\max\;(\text{Gy})$	0.85 (0.13, 1.48)	0.48 (0.05, 0.81)
${\text{PTV}}_{-}\text{mean}\;(\text{Gy})$	$0.35{\left(0.27,0.46\right)}^{a}$	$0.39{\left(0.30,0.53\right)}^{a}$
${\text{PTV}}_{-}V_{100\%}\;(\%)$	1.7 (1.0, 3.2)	$1.9{\left(1.3,2.5\right)}^{a}$
${\text{PTV}}_{-}D_{90\%}\;(\text{Gy})$	0.22 (0.05, 0.35)	$0.26{\left(0.13,0.44\right)}^{a}$
${\text{CORD}}_{-}V_{10\%\text{Gy}}\;{\left(\%\right)}^{b}$	7.8 (4.4, 14.4)	$7.5{\left(4.5,12.3\right)}^{a}$
${\text{CORD}}_{-}\max\;(\text{Gy})$	$0.55{\left(0.33,0.85\right)}^{a}$	$0.58{\left(0.35,0.69\right)}^{a}$
${\text{CORD}}_{-}D_{0.1\text{CC}}\;(\text{Gy})$	$0.61{\left(0.36,0.71\right)}^{a}$	$0.63{\left(0.49,0.71\right)}^{a}$

a p‐value <0.01 Note that the p‐values for all other endpoints were <0.05.

b ${\text{CORD}}_{-}V_{12\text{Gy}}$ was used for the sacrum case.

## IV. DISCUSSION

We performed a comprehensive dosimetric evaluation of O‐MAR algorithm on spine SBRT patients with spinal prostheses and a phantom that mimics the clinical situations. Both the phantom and patient studies showed that the O‐MAR algorithm reduced metal artifacts and provided better visualization of the anatomical structures and spinal prostheses. The phantom study demonstrated that O‐MAR reduced the original severe streaks around the titanium insert, but it introduced some dispersed artifacts in other directions. This is a well‐known drawback of filtered back‐projection–based metal artifact reduction algorithms resulting from removing and interpolating projection data. From the patient study, all five cases showed some degree of image quality improvement from O‐MAR correction. The cases with small artifact reduction (Fig. 3(c) and (d)) had smaller metal implants compared to the cases with large artifact reduction (Fig. 3(a) and (b)), suggesting the efficacy of O‐MAR reduced with decreasing size of implants. Furthermore, some residual artifacts remained in the O‐MAR–corrected images, similar to what was observed in previous O‐MAR studies.^(17)^ There are two likely causes for the residual artifacts: imperfect projection modification algorithm and photon scattering around the metal implant.

For the dosimetric comparison on the phantom images, two scenarios were investigated where the titanium insert was placed either inside or outside of the spinal tumor. Only one small discrepancy was found in the DVHs: the DVH of the PTV for the reference image was slightly different from the DVHs of the PTVs for the non‐O‐MAR and O‐MAR images when the titanium was inside of the tumor. This discrepancy is probably due to more metal artifacts in the PTV when the titanium was inside of the tumor compared to outside of the tumor. In addition, the high gamma index (>99.96%) between non‐O‐MAR and O‐MAR phantom images confirmed that the dose distributions were very similar.

Dosimetric comparison for the patient images was carried out via both the pencil beam and Monte Carlo methods in the iPlan TPS. The DVHs demonstrated that there were no significant dosimetric differences in the PTV and spinal cord between the non‐O‐MAR and O‐MAR image sets. The high gamma index with the criteria of 2%/2 mm (>99.3% for pencil beam and >98.6% for Monte Carlo) between non‐O‐MAR and O‐MAR patient images also demonstrated the minimal impact of O‐MAR on dose distributions. Note that, with the stricter criteria of 1%/1 mm, there was a greater difference in the gamma index values between pencil beam (>96.9%) and Monte Carlo (>95%). Quantitative analysis showed that the dosimetric differences between non‐O‐MAR and O‐MAR were small when the same dose calculation method was used. For the pencil beam method, the largest differences were that O‐MAR decreased ${\text{PTV}}_{-}\max$ by 0.13 Gy, decreased ${\text{PTV}}_{-}V_{100\%}$ by 0.9%, and increased ${\text{CORD}}_{-}V_{10\text{Gy}}$ by 0.9%. For the Monte Carlo method, the largest differences were that O‐MAR decreased ${\text{PTV}}_{-}\max$ by 0.79 Gy and decreased ${\text{CORD}}_{-}V_{10\text{Gy}}$ by 1.2%. Nonetheless, all clinical dose constrains were met using the O‐MAR dataset, compared to the non‐O‐MAR dataset. This indicates that O‐MAR correction does not impact the dose calculation when the same dose calculation method is used. However, different dose calculation methods would yield different dose distributions even when the same dataset was used. In this study, the pencil beam method was shown to underestimate the dose constraints compared to the Monte Carlo method (Table 2). For example, some patient plans would exceed the dose constraint for ${\text{CORD}}_{-}V_{10\text{Gy}}$ when calculated with Monte Carlo method even though they were within the constraint when calculated with the pencil beam method. Therefore, choosing an appropriate dose calculation method for spine SBRT cases is critical to meet the clinical constraints. The results of this study will be incorporated in the future outcome study for patients with spine prostheses receiving SBRT.

Overall, O‐MAR had little effect on dosimetry, regardless of the metal location and dose calculation methods. The insignificant effects of O‐MAR on dosimetry may be due to two factors: the type of metal and the size of implants. Some recent studies suggested that the impact of MAR on dosimetry is dependent on the atomic number of the metal: low‐Z metals, such as titanium (Z=22), do not produce significant dose errors; whereas high‐Z metals, such as platinum (Z=78) and gold (Z=79), substantially affect the dose calculation.^26^, ^27^ This helps explain the minimal dosimetric differences observed in this study between non‐O‐MAR and O‐MAR image sets, since the spinal prostheses were all made of titanium. For the same type of metal, the size of implants determines the degrees of artifacts with larger implants produce more severe artifacts.^(28)^ Because of the insignificant dosimetric effects of O‐MAR on spine SBRT patients with spinal prostheses, current clinical dose constraints for targets and OARs can be applied to the O‐MAR–corrected images without further modifications. Compared to non‐O‐MAR images, O‐MAR–corrected images can be safely used for clinical treatment planning. The reduction of metal artifact in the planning CT can also improve alignment accuracy for image‐guided radiation therapy.

This study has a few limitations. First, the patient population is relatively small. This is due to the limited number of patients with spine prostheses. Second, we used different TPSs for the phantom and patient studies. iPlan was utilized at our institution to treat the spine SBRT patients selected for this study. It would be ideal to use iPlan on the phantom study, as well. However, iPlan is not capable of overriding density for individual structures. We chose Pinnacle for the phantom study because it can override the density, and the accuracy of its collapsed cone convolution algorithm was considered similar to Monte Carlo calculation.^(29)^

## V. CONCLUSIONS

For spine SBRT patients with spinal prostheses, O‐MAR image reconstruction can reduce metal artifacts without affecting dose calculation accuracy. The O‐MAR–corrected image is not only a better planning CT for contour delineation, but also a high‐quality reference image for image guidance. Therefore, O‐MAR–corrected images are recommended for radiotherapy treatment planning on spine SBRT patients with spinal prostheses.

## ACKNOWLEDGMENTS

This work was supported by a research grant from Philips Healthcare Inc. (Cleveland, OH). The authors would also like to acknowledge an iPlan workstation provided by BrainLAB Inc. (Westchester, IL).
